# Bidirectional Transfer of RNAi between Honey Bee and *Varroa destructor*: *Varroa* Gene Silencing Reduces *Varroa* Population

**DOI:** 10.1371/journal.ppat.1003035

**Published:** 2012-12-20

**Authors:** Yael Garbian, Eyal Maori, Haim Kalev, Sharoni Shafir, Ilan Sela

**Affiliations:** The Hebrew University of Jerusalem, The Robert H. Smith Faculty of Agriculture, Food and Environment, Rehovot, Israel; Stanford University, United States of America

## Abstract

The mite *Varroa destructor* is an obligatory ectoparasite of the honey bee (*Apis mellifera*) and is one of the major threats to apiculture worldwide. We previously reported that honey bees fed on double-stranded RNA (dsRNA) with a sequence homologous to that of the Israeli acute paralysis virus are protected from the viral disease. Here we show that dsRNA ingested by bees is transferred to the *Varroa* mite and from mite on to a parasitized bee. This cross-species, reciprocal exchange of dsRNA between bee and *Varroa* engendered targeted gene silencing in the latter, and resulted in an over 60% decrease in the mite population. Thus, transfer of gene-silencing-triggering molecules between this invertebrate host and its ectoparasite could lead to a conceptually novel approach to *Varroa* control.

## Author Summary

Acquisition of RNAi components (dsRNA, siRNA) by ingestion and their spread within the recipient organism has been previously reported by us and others. Here we extend such observations, demonstrating cross-species horizontal transmission of dsRNA which, upon transmission from one organism to another still retains its biological activity. We show that dsRNA ingested by honey bees is further transmitted to the parasitic mite *Varroa destructor* that feeds on the honey bee's hemolymph. Reciprocally, dsRNA-carrying *Varroa* transmits the dsRNA back to bees. Furthermore, we demonstrate that bees ingesting dsRNA of *Varroa* gene sequences become vectors of dsRNAs, transmitting the signals to the *Varroa*, thus engendering silencing of mite genes and resulting in a significant phenotype, *Varroa* mortality. The exchange of active silencing signals between the honey bee and the mite suggests a potential RNA-based interaction between invertebrate hosts and parasites. Furthermore, our results offer a potentially conceptually new control measure for the mite *Varroa destructo*r, which is one of the greatest threats to apiculture.

## Introduction

The European honey bee, *Apis mellifera*, plays a key role in pollination. One third of the world's food crops as well as many wild plants depend on honey bees for pollination [1]. In recent years, honey bee colonies suffer from severe losses worldwide. Pests and pathogens are mainly involved in the decrease of honey bees vitality and colony losses including: mites, viruses, fungi, bacteria, and other insects [2]. However, the mite *Varroa destructo*r is considered one of the greatest threats to apiculture, not only because of its direct deleterious effect, but also by being a vector of several important bee viruses [3]–[8]. *Varroa destructor* is an obligatory parasite that feeds on the hemolymph of developing and mature honey bees. *Varroa* mites invade cells of bee larvae just before they are sealed, feed on the hemolymph of the developing bee and proliferate there. When the adult bee emerges, the attached female mites emerge with it. They may then transfer to another bee or another bee larval cell [3], [9]. Without proper treatment, honeybee colonies infested with *Varroa destructor* typically collapse within 2 to 3 years [3], [10]. Beekeepers use chemicals such as the organophosphate coumaphos, tau-fluvalinate and the formamidine amitraz to control *Varroa*, but the mites evolve resistance to such chemicals [11]–[14]. Therefore, alternative measures for *Varroa* control are sought, such as breeding bees for tolerance [3]. Here we report that *Varroa* gene expression can be modulated by RNA interference (RNAi) mediated by the bees, which may lead to a potential new conceptual approach to *Varroa* control.

RNAi is an RNA-mediated sequence specific gene-silencing mechanism [15]. RNAi has been demonstrated to moderate gene expression in a wide variety of organisms including plants, mammals, insects and ticks [16], [17]. The silencing pathway is initiated by the presence of endogenous or exogenous double-stranded RNAs (dsRNAs) that is then cleaved by RNase III-like enzymes resulting in small (21–26 bp) interfering RNAs (siRNA). SiRNAs guide protein complexes to RNAs carrying homologous sequences and target the RNA for degradation, or RNA-directed DNA methylation or chromatin remodeling [16]. Recently, transfer of RNAi from plants to insects and nematodes has been reported [18]–[21] as well as vertical transgenerational transfer of silencing signals [22]. RNAi affecting a germline has also been reported very recently [23]. DsRNA-mediated gene knockdown has also been demonstrated in *Varroa* by soaking the mite with dsRNA-containing solution [24]. In honey bees, ingestion of dsRNA has been successfully used to investigate gene function as well as for direct application against viral infection and the endoparasite *Nosema ceranae* [25]–[31].

Our previous indications of the effectiveness of ingested dsRNA-mediated silencing in bees [32] suggested that dsRNA is spread systemically in treated bees. We therefore hypothesized that ingested and systemically spread dsRNA might be horizontally transferred from treated bee to *Varroa*, in which case the bees could serve as RNAi vectors. Here we demonstrate reciprocal horizontal transfer of dsRNA ingested by honey bees to *Varroa* mites and then on to *Varroa*-parasitized bees. A significant phenotype, a measure of *Varroa* control, was achieved by RNAi that was vectored through bees to silence *Varroa*-specific genes.

## Results

### Direct and indirect horizontal transfer of dsRNA between bees and *Varroa* mites

To determine whether dsRNA is transferred from honey bees to *Varroa*, initial studies were carried out with biologically irrelevant dsRNA marker carrying a segment of the gene for green fluorescent protein (GFP). The use of dsRNA-GFP (Table S1) potentially reduced endogenous sequence “noise” and minimized possible silencing of bee or *Varroa* gene expression (the list of dsRNA sequences used throughout this study is shown in Table 1 and the actual sequences are depicted in Table S1). Since *Varroa* mites suck substantial amounts of hemolymph from both adult and developing bees in sealed brood cells, we tested whether dsRNA is horizontally transferred in both cases. Adult bees were fed a sucrose solution containing dsRNA, resulting in direct transfer of dsRNA from adult bees to phoretic mites feeding on their hemolymph. Adult nurse bees produce jelly that they feed to developing larvae in brood cells, prior to sealing the cells: here, the transfer of dsRNA from a developing bee in a sealed cell to mites feeding on their hemolymph is indirect.

**Table 1:** List of *Varroa* dsRNA sequences used in *Varroa* gene silencing.

Used in mixture:	Corresponding gene function similar to:	Sequence number
I and II	α-tubulin	#1
II	α-tubulin	#2
II	α-tubulin	#3
I and II	RNA polymerase III	#4
II	RNA polymerase III	#5
II	RNA polymerase II	#6
II	RNA polymerase I	#7
I and II	Vacuolar translocating ATPase	#8
II	Vacuolar proton ATPase	#9
II	Na+/K+ ATPase	#10
II	Apoptosis inhibitor IAP	#11
I and II	Apoptosis inhibitor FAS	#12
II	Apoptosis inhibitor iap1 and iap2	#13
I and II	Apoptosis inhibitor iap1 and iap2	#14

This table lists the *Varroa* dsRNA sequences numbers, genes function, and the mixtures they are contained in. The full sequences are located in Table S1.

**Figure 1:**
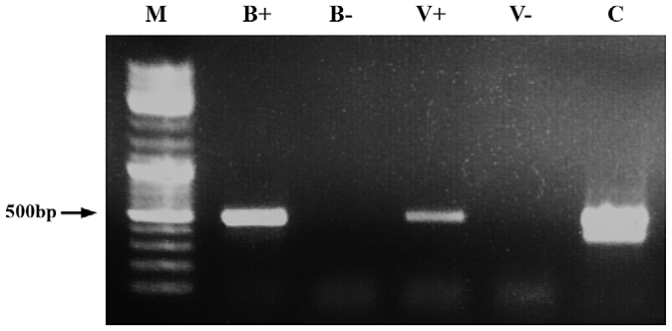
Demonstration of dsRNA transmission from adult bee to *Varroa*. RT-PCR was performed on RNA extracted from a bee that had ingested dsRNA-GFP (B+) and from an untreated bee (B−). V+ and V− represent amplification of RNA extracted from *Varroa* parasitizing dsRNA-GFP-treated bees and untreated bees, respectively. M = size markers. C = positive control (GFP-carrying plasmid).

To test for direct horizontal transfer, we placed 30 worker bees in plastic containers, and fed them with dsRNA-GFP in a 50% sucrose solution for 8 days. *Varroa* mites were introduced to the containers on the fifth day of feeding. After 3 days, *Varroa* that were attached to the bees were removed. Transfer of dsRNA from bees to mites was determined by RT-PCR of templates extracted from the mites and the presence GFP sequence in the mite indicated bee-to-mite transfer (Figure 1).

**Figure 2:**
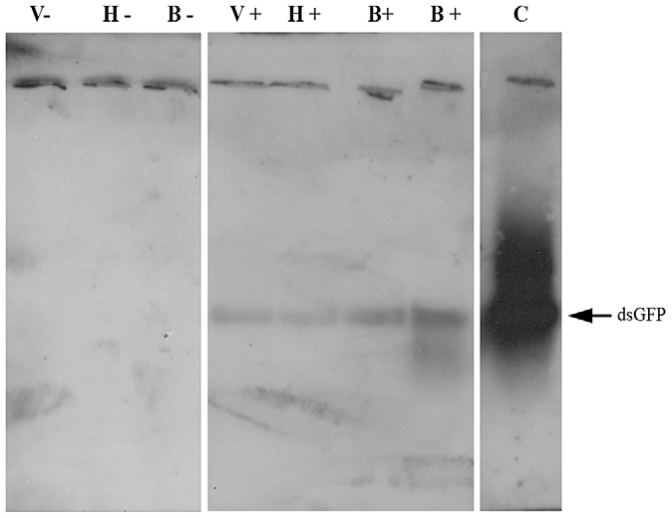
Demonstration of dsRNA transmission from adult bee to *Varroa* via the bee hemolymph. Northern blot assay was performed on RNA extracted from a bee that had ingested dsRNA-GFP (B+) and from an untreated bee (B−), pooled RNA extracted from hemolymph collected from bees that had ingested dsRNA-GFP (H+) and from untreated bees (H−), and pooled RNA extracted from *Varroa* mites parasitizing dsRNA-GFP-treated bees (V+) and untreated bees (V−). C = positive control (GFP-carrying plasmid).

Mites can absorb dsRNAs and siRNAs by physical contact [24]. To determine any biological function, it was necessary to establish that RNAi triggers were delivered by food ingestion and not by accidental contamination, and that the acquired dsRNA was present in the bee hemolymph (the food source of *Varroa*). To this end we strapped individual bees to a hollow plastic tube, and fed them directly to their proboscis with dsRNA-GFP. This prevented potential contamination of other bee parts by self or mutual grooming with other bees. On the following day, we released each bee to a separate plastic box, and added two female *Varroa* mites to each of the above boxes. The *Varroa* were collected from a remote hive that had not been exposed to dsRNA. On the third day, we collected *Varroa* attached to the bees, and extracted hemolymph from the bees. DsRNA-GFP was detected in RNA extracted from hemolymph of dsRNA-GFP-treated bees. DsRNA-GFP was also found in *Varroa* parasitizing the treated bees (Figure 2), indicating that dsRNA transfer was *via* ingestion and that treated bees do carry dsRNA in their hemolymph.

**Figure 3:**
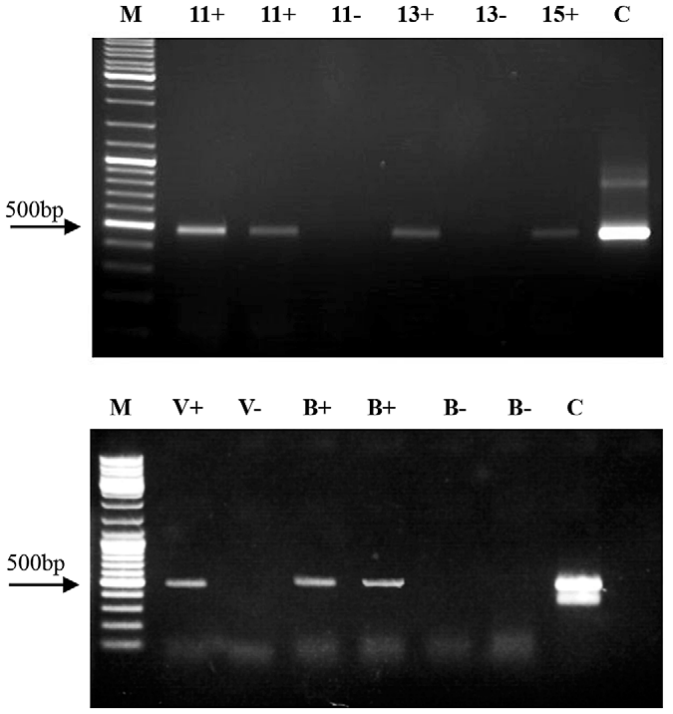
Demonstration of dsRNA transfer from bee to *Varroa* and from *Varroa* to bee. (A) Indirect dsRNA transmission from bee to *Varroa* parasitizing bee brood. RT-PCR of *Varroa*-extracted RNA. Numbers represent days from the beginning of dsRNA feeding. M = size markers. C = positive control (GFP-carrying plasmid). + indicates samples collected from dsRNA-GFP-treated hives. – indicates samples collected from untreated, control hives. (B) DsRNA transmission from *Varroa* to bee. RT-PCR was performed on RNA extracted from a dsRNA-GFP-carrying *Varroa* (V+) and from *Varroa* devoid of dsRNA-GFP (V−). B+ represents amplification of RNA from bees infested with dsRNA-GFP-carrying *Varroa* and B– represents amplification from bees infested with dsRNA-GFP-devoid *Varroa*. M and C: as in legend for A.

Pursuant to establishing a hemolymph-mediated RNAi transfer we studied the possibility of indirect horizontal RNAi transfer. We placed ca. 250 worker bees and a laying queen in mini-hives and fed them dsRNA-GFP for 8 days. *Varroa* mites were introduced into the mini-hives on the fifth day of feeding, and were later collected from sealed larval/pupal cells. The presence of dsRNA-GFP in *Varroa* mites collected from sealed cells on various days of larval/pupal development indicated indirect transfer of dsRNA from bee larvae (which were fed by nurse bees that had themselves fed on dsRNA-containing sugar solution) to *Varroa* (Figure 3A).

### Bidirectional horizontal transfer of dsRNA between bees and *Varroa* mites

Having shown direct and indirect transfer of dsRNA from bees to *Varroa*, we wanted to test whether a mite that has acquired dsRNA from a bee can then transfer it to another bee that it parasitizes. In the direct transfer experiment, bees were fed dsRNA-GFP in 50% sucrose solution for 8 days, and *Varroa* mites were added on the fifth day of feeding. To test for bidirectional horizontal transfer, on the eighth day the mites were removed from the dsRNA-carrying bees and introduced into a container with untreated bees for 4 days. DsRNA-GFP was detected in RNA extracts of bees that had not consumed dsRNA, but were parasitized by *Varroa* mites that previously parasitized dsRNA-carrying bees (Figure 3B). The presence of dsRNA-GFP in the parasitized bees indicated direct reciprocal transfer of dsRNA from bee to *Varroa* and on to another bee.

#### DsRNA stability in hives

We tested the stability of dsRNA under honey bee hive conditions in order to determine the potential of dsRNA for *Varroa* control. We placed a cage with 10 bees in an empty second floor of a hive, separated by screen from the populated bottom floor, so that the caged bees experienced an environment similar to that of the hive. The caged bees had access to a feeder containing dsRNA-GFP in 10 ml 50% sucrose solution, identical to the dsRNA-GFP concentration in the other experiments. Under these conditions, dsRNA-GFP slowly degraded along the first three days. By day six, dsRNA-GFP was almost fully degraded (Figure S1).

### Silencing of *Varroa* gene expression mediated by bees ingesting dsRNA

Once we had established horizontal transfer of physiologically inert dsRNA between bees and *Varroa*, we explored the possibility that RNAi that originated in the bee might affect gene expression in the mite.

When this study was initiated, the *Varroa* genome had not been elucidated (recently, partial *Varroa* genome information has been released [33]). Therefore, we designed a number of genes whose silencing was expected to harm the *Varroa* mite. We chose fundamental housekeeping genes involved in cytoskeleton assembly, energy transfer and transcription. In addition, we chose genes involved in apoptosis inhibition (assuming that their silencing would enhance apoptosis). We aligned several published mite and insect gene sequences and determined conserved regions (Figure S2). We designed probes to the conserved regions, screened a *Varroa* cDNA library and isolated the respective *Varroa* genes. The *Varroa* dsRNA sequences selected for *Varroa* gene silencing are presented in Table S1. To prevent off-target human or bee gene silencing, these sequences did not correspond to any *A. mellifera* or human genes (Table S2). In some cases, sequences from the same gene family were selected. The dsRNAs of the selected sequences were prepared as previously described [29].

**Figure 4:**
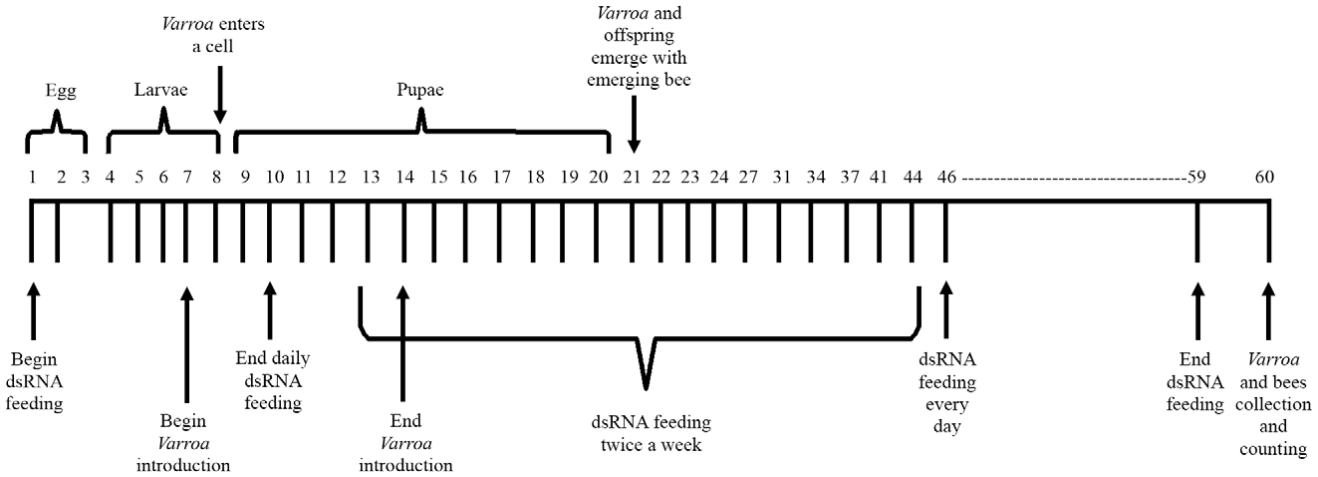
Schematic representation of the honey bee feeding regimen. The experiment lasted for 60 days. Top: schematic representation of a honey bee's life cycle. Bottom: experimental procedures on a timescale.

**Figure 5:**
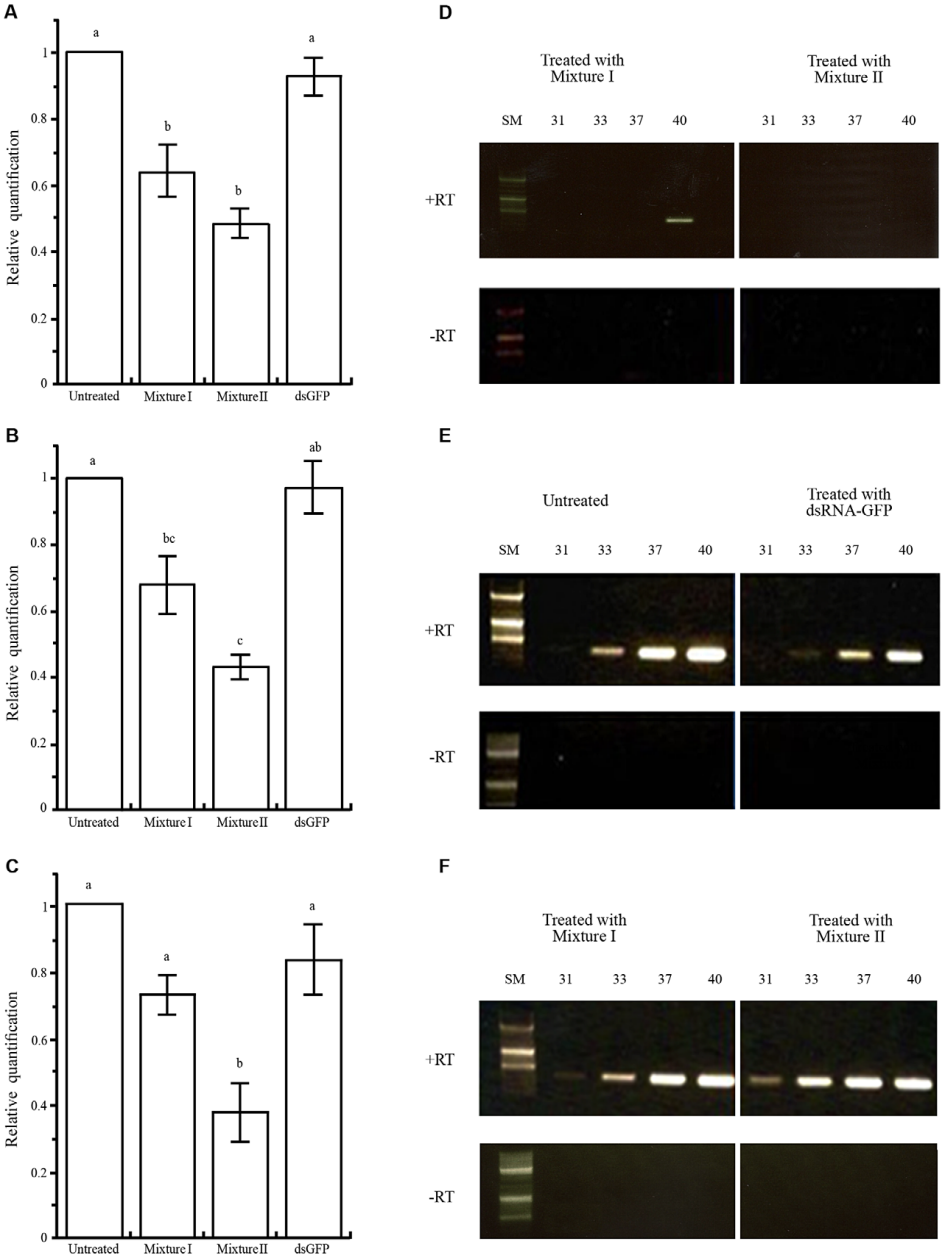
Silencing of *Varroa* gene expression following horizontal transfer of dsRNA from bee to *Varroa*. (A–C) Results (mean ± SE) of real-time RT-PCR of *Varroa* genes 4, 14 and 9, respectively (Table S1). RNA from *Varroa* infesting untreated bees, which did not ingest dsRNA, or those that ingested the physiologically inert dsRNA-GFP served as controls. Note that treatment I is devoid of sequence 9. Different letters above columns indicate significant differences between treatments (*P*<0.05). Details of the RT-PCR assays and statistical analysis are described in the Materials and Methods. (D–E) Semi-quantitative RT-PCR for expression of the apoptosis inhibitor FAS gene (sequence 12, Table S1). RNA was extracted from *Varroa* fed on bees that had ingested Mixture I or II (D). RNA was extracted from *Varroa* fed on dsRNA-untreated bees, and on bees that had ingested dsRNA-GFP (E). (F) Amplification of actin as a standardizing internal control. Reactions devoid of reverse transcriptase (RT) served as controls for the absence of DNA contamination. Number of PCR cycles is indicated at the top of each electropherogram. SM = size markers.

To determine whether *Varroa* gene silencing can be mediated via bees that have ingested dsRNA, we placed ca. 250 worker bees and a laying queen in mini-hives. We prepared two mixtures of the *Varroa* dsRNA: Mixture I contained sequences derived from five *Varroa* gene sequences (sequences 1, 4, 8, 12, and 14 described in Tables 1 and S1) and Mixture II contained all 14 *Varroa* gene sequences (Tables 1 and S1). Mini-hives fed with Mixture I or II each served as a treatment group. In addition, mini-hives fed with dsRNA-GFP or only sucrose solution served as two control groups. After 1 week of feeding, we introduced *Varroa* mites every day for a week (Figure 4). At the end of the 60-day experiment, we sampled *Varroa* mites from all four treatment groups and determined transcript levels of four selected *Varroa* genes by real-time RT-PCR (sequence 4, homologous to RNA polymerase III; sequence 9, homologous to vacuolar proton ATPase; and sequence 14, homologous to apoptosis inhibitor iap1 and 2) or semi-quantitative RT-PCR (sequence 12, homologous to apoptosis inhibitor FAS). The results confirmed that the dsRNA fed to the bees indeed engendered gene silencing in the parasitic *Varroa* mites, inhibiting expression levels of the tested genes by approximately 35 to 60% (Figure 5). The transcript levels of the three genes that we analyzed by real-time RT-PCR from Mixture II (genes 4, 14, and 9) were significantly lower relative to the untreated and the dsGFP controls (Figure 5, A–C, respectively). The reduction in transcript levels of genes 4 and 14 did not differ significantly between Mixtures I and II. However, in Mixture I there was a trend for higher transcript levels, especially of the latter gene. Mixture I did not contain sequence 9, and consistently transcript level of this gene was not affected by Mixture I relative to the untreated and the dsGFP controls. Both mixtures contained the sequence for *Varroa* gene 12, and semi-quantitative RT-PCR showed greatly reduced expression of this gene by both mixtures (Figure 5D) relative to untreated bees and to bees treated with dsRNA-GFP (Figure 5E), and to expression of actin as a standardizing internal control (Figure 5F).

### Reducing *Varroa* population in hives following bee-mediated gene silencing of *Varroa* gene expression

**Figure 6:**
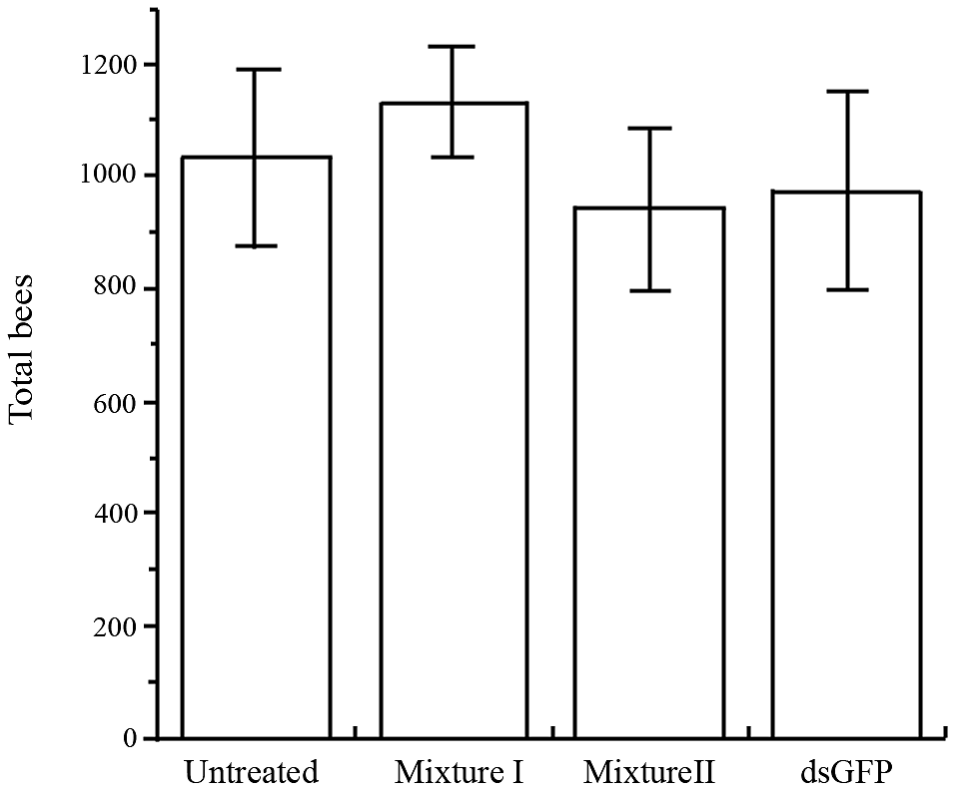
Mean (+ SE) total number of bees (capped brood and adults) in four treatments. Treatments did not differ significantly.

Once we had demonstrated silencing of several *Varroa* genes, we proceeded to monitor mite survival. First, we tested whether the dsRNA mixtures affect bee survival by counting all mature bees and sealed brood in the mini-hives at the end of the experiment. Bee population size did not differ between control and dsRNA-treated mini-hives (F_3,29_ = 0.62, *P* = 0.608; Figure 6). The results were similar when brood and adult bees were analyzed separately (not shown). Hence, the dsRNA mixtures were not deleterious to bees, indicating no off-target effect.

We proceeded to investigate whether bee-mediated silencing of *Varroa* genes could reduce the size of *Varroa* population in infested hives. We determined the number of *Varroa* individuals per bee by examining the mite population on mature bees and in sealed brood cells at the end of the experiment.

**Figure 7:**
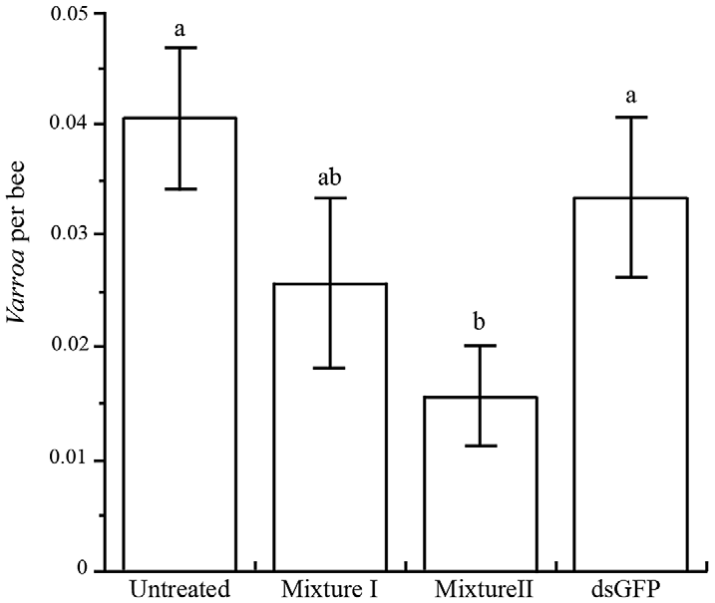
Mean (+ SE) number of *Varroa* mites per bee in four treatments. Different letters above columns indicate significant differences between treatments (*P*<0.05).

*Varroa* infestation was reduced in mini-hives treated with *Varroa* dsRNA compared to the controls (F_3,29_ = 5.65, *P* = 0.0035; Figure 7). The effect was greater with Mixture II, which targeted more genes than Mixture I, reducing *Varroa* populations by an average 53% compared to the dsRNA-GFP control, and by 61% compared to the untreated control.

## Discussion

Gene silencing following ingestion of dsRNA by honey bees has been previously reported [25]–[32]. Here, we show that the ingested dsRNA can be delivered across species to a bee-hemolymph-dependent parasite. This RNAi transfer may cause silencing of *Varroa* gene expression and reduce mite populations in hives. Quantitative and semi-quantitative RT-PCR indicated that the dsRNAs affected the targeted *Varroa* genes. From an RNA biology point of view, the possibility that closely-associated organisms may interact via RNAi pathways should be further explored.

The *Varroa* mite has been demonstrated to be a vector for bee viruses [2], [6]–[8]. Similarly, we show that the mite can also vector RNAi-triggers, which were acquired from hemolymph of bees that had consumed dsRNA. Additionally, we demonstrated that bees vector biologically active dsRNA to the *Varroa* mite. The occurrence of such reciprocal interactions raises the hypothesis that bees may be potential vectors for *Varroa*-affecting viruses.

Although the main emphasis of this study is the cross-species transfer and effect of RNAi, it also reflects on a promising concept of *Varroa* control. With *Varroa* mites evolving resistance to the chemicals used for their control [11]–[14], our study provides a novel potential approach for relieving the most serious economic burden on apiculture. Consistent with our findings of the stability of dsRNA in the honey bee colony, it is stable enough to be efficient when administered to the colony in sugar solution [29], [32], yet it eventually degrades in an environment favorable to bacterial growth. *Varroa* mainly devastate colonies when *Varroa* population grows unchecked. Effective control measures do not necessarily need to completely eradicate the *Varroa* population [34], [35]. Further studies would need to monitor the effect of dsRNA treatment on *Varroa* population dynamics and long-term effect on honey bee colonies under field conditions. Furthermore, the dsRNA formula may be optimized by finding more vital target genes that will lead to a greater reduction of the mite population in hives with less amount of dsRNA fed to infested hive.

We prepared two dsRNA formulations: Mixture I targeted 5 *Varroa* gene sequences and Mixture II targeted 14 *Varroa* gene sequences. Mixture II tended to reduce *Varroa* populations more effectively (Figure 7). This suggests the existence of overlapping metabolic pathways or that some of the gene products are stable and remain active even though their respective gene's expression had been silenced.

We selected dsRNA sequences that are not homologous to honey bee (or human) sequences. As in other reports [31], [32], silencing (in this case of the *Varroa* genes) did not affect the vigor of the bees (Figure 6). Notably, we did not notice the off-target effects reported by Jarosch and Moritz [36]. Although *Varroa* infestation was greater in control vs. treatment mini-hives (Figure 7), this did not affect the strength of the hives at the end of our experiment (Figure 6). This is not surprising, since *Varroa* were present in our hives for only about 7 weeks. *Varroa* damage is cumulative and is minimal in newly infested hives (hives collapse after 2–3 years [3], [10]).

## Materials and Methods

### DsRNA preparation

DsRNA was prepared according to Maori et al. [29]. Sequences were amplified by PCR using specific primers including the 5′ tail of the T7 promoter (Table S1). PCR products were TA cloned into the plasmid pDRIVE and sequenced. Amplicons were used as template for in-vitro transcription.

### RNA extraction and analysis

Total RNA for dsRNA-GFP detection experiments was isolated from a single honey bee or from 10 *Varroa* mites, using phenol-chloroform extraction (peqGOLD Trifast, Peqlab). Total RNA for *Varroa* dsRNA experiments or for dsRNA-GFP detection was isolated from 5 *Varroa* mites or from 50 mites, respectively, with the ZR Tissue & Insect RNA MicroPrep (Zymo Research) according to the manufacturer's instructions. Eluted RNA was treated with TURBO DNA-free kit (Ambion, Austin, TX, USA) and tested for DNA contamination. *Varroa* RNA was then co-precipitated with glycogen and 3 M sodium acetate in 70% ethanol and resuspended in 20 µl of RNAse-free water. The amount and quality of the RNA samples were determined by spectrophotometer (NanoDrop Technologies, Wilmington, DE, USA). RNA from hemolymph was extracted using the phenol: chloroform: Isoamyl alcohol method according to published protocol [37].

### DsRNA-GFP detection by RT-PCR

DsRNA-GFP was detected by RT-PCR using Verso 1-Step RT-PCR (Thermo Scientific) with specific GFP primers according to the manufacturer's protocol. Total RNA samples extracted from 10 *Varroa* or 1 honey bee were used as templates.

### DsRNA-GFP detection by northern blot analysis

Northern blot assay for detecting dsRNA-GFP was performed as follows: Samples of RNA (500 ng) were electrophoresed on 1.2% agarose gel. The gel was washed with 0.25 M HCl, denaturation solution, neutralization solution and 10XSSC before the RNA was transferred to a positively charged nylon membrane (Roche Diagnostics). The membrane was treated according to the manufacturer's protocol, with DIG-labeled probe (Roche Diagnostics) of GFP sequence corresponding to the sequence used as template for dsRNA-GFP synthesis.

### cDNA library construction

A *Varroa* cDNA library was prepared using a Smart cDNA construction kit (Clontech) according to the manufacturer's instructions. Genes involved in four activity categories were designated. Database-recorded mite and insect genes belonging to those groups were aligned (Figure S2). Conserved sequences were determined for each group and served as probes for selecting the homologous genes from a *Varroa* cDNA library. The actual *Varroa* genes were sequenced. Segments of *Varroa* genes, 200 to 450 bp in length, which did not correspond in sequence to any bee or human genes (identity of less than 21 consecutive bases; Table S2), were selected. The selected *Varroa* sequences and GFP partial sequence are presented in Table S3.

### Gene expression: Real-time RT-PCR and semi-quantitative RT-PCR

RNA (400 ng) was subjected to reverse transcription with random hexamers using the Verso cDNA synthesis kit (Thermo Scientific). Each sample of the obtained cDNA was diluted 1∶50 before amplification. Real-time quantitative PCR was performed by LightCycler 480 (Roche) and was analyzed with the instrument's software. The employed primers and probes are listed in Table S4. The real-time PCR program was as follows: 95°C for 10 min, followed by 45 cycles of 95°C for 10 s and 60°C for 30 s. At the end, samples were subjected to 40°C for 30 s. 18S rRNA was used as an internal control for the standardization of RNA levels.

The semi-quantitative PCR program was as follows: 95°C for 10 min, followed by 40 cycles, each consisting of 95°C for 10 s and 65°C and 55°C for 30 s for the apoptosis inhibitor (FAS) and its internal standardization control (actin), respectively, followed by 72°C for 30 s. Every three cycles starting from cycle 31 for FAS and 29 for actin, a tube was taken out, incubated for 5 min at 72°C and stored at −20°C. Samples were analyzed on a 1.2% agarose gel. Each qPCR experiment was repeated three times.

### Regimen of dsRNA-GFP feeding

To test for direct transfer of dsRNA-GFP from adult bee to mite, 1-day-old bees that emerged in an incubator were placed in four plastic containers (30 bees per container). Two containers were fed with 30 µg dsRNA-GFP in 200 µl of 50% sucrose solution for 8 days, and the other two containers were controls, fed only 50% sucrose solution. Adult female *Varroa* (n = 30) were introduced into each container on day 5. After 3 days, *Varroa* that were attached to bees were removed and collected and their RNA was isolated for dsRNA-GFP analysis. A possible caveat of this experiment was that dsRNA-GFP would be transferred to *Varroa* through direct contamination with dsRNA-GFP in the containers, or from contact with contaminated bee body parts.

We therefore performed an additional experiment to test for dsRNA transfer to *Varroa* mites via the bee hemolymph. In the morning of the experiment, 150 bees were collected from the area of combs containing open larvae; these tend to be nurse bees. Each bee was strapped into a hollow plastic tube in a manner that ensured their ability to extend their proboscis, and minimized injuries of the bees upon release [38]. The bees were divided into two groups: Individuals in the first group were fed with 2.5 µg dsRNA-GFP in a 5 µl 50% sucrose solution. Bees in the second group served as the control group, and were fed with 5 µl 50% sucrose solution only. In order to avoid contamination of dsRNA-GFP on the bee body, the sucrose solution was given directly to the proboscis. In addition, in order to prevent starvation of the bees overnight, both groups (treated and control) were fed 5 µl 50% sucrose solution in the evening. The following day, each bee was released gently from the hollow plastic tube, placed in a clean cage and supplied with candy (67% sugar powder and 33% honey). Two female adult *Varroa* mites were added to each of the above bees. *Varroa* were collected from a mite-infested hive that has never been exposed to dsRNA. On the third day, each bee was anaesthetized on ice, and 1–10 µl of hemolymph was collected. The collection of hemolymph was performed by pricking a hole in the inter-segmental membrane between the 2nd and 3rd abdominal segment, and inserting a capillary tube. Prior to hemolymph collection, *Varroa* mites, which were attached to the bee's body, were collected. All samples (*Varroa* mites, hemolymph and bees) were placed directly on ice, and then stored at −80°C for molecular analysis.

To test for bidirectional transfer of dsRNA-GFP from bee to mite and on to another bee, some of the *Varroa* that had been detached from bees were transferred to containers with newly emerged, untreated bees for 4 days and their RNA was isolated for dsRNA-GFP analysis. Every day, bees in all containers were given an additional 1 ml sucrose solution after finishing their treatment. In addition, bees had free access to a pollen patty consisting of 70% pollen mixed with sugar powder.

To test for indirect transfer of dsRNA-GFP from adult bee to bee larva and on to mite feeding on the hemolymph of the developing bee in a sealed cell, a cup of bees (ca. 250) and a laying queen were introduced into each mini-hive (two replicates in each of two enclosures). DsRNA-GFP (200 µg per hive) was provided daily in 5 ml 50% sucrose solution for 8 days. Thirty *Varroa* mites were introduced to the hives on the fifth day. Adult female *Varroa* were collected from sealed cells from day 11 till day 30 and their RNA was isolated for dsRNA-GFP analysis.

### DsRNA-GFP stability in 50% sucrose solution

To test the dsRNA stability in the hive, we placed 10 bees in a cage, and exposed them to 10 ml of 50% sucrose solution that contained 200 µg dsRNA-GFP. Hence, dsRNA-GFP final concentration was 20 ng/µl, identical to the dsRNA-GFP concentration that was used in the other experiments. The cage was placed in a vacant second floor of a hive, separated by a screen from the populated bottom floor. Thus the caged bees were exposed to the hive's environment. Samples from the sugar solution were taken on days 1, 2, 3 and 6, and placed on ice. The caged bees died on day 2, possibly due to a heat wave. Sucrose concentration in the samples was determined with a refractometer, and if needed, equilibration with water was done. Samples were then stored at −80°C until analysis. Samples were analyzed on 1.2% agarose gel, loaded with 5 µl from each sample (100 ng of dsRNA-GFP at time zero).

### Regimen of honey bee feeding with *Varroa* dsRNA

The experiment with *Varroa* dsRNA was conducted in mini-hives, 12 mini-hives per replicate, and was repeated three times. In each replicate, a cup of bees and a laying queen were placed in each mini-hive. Three mini-hives were randomly assigned to one of four netted enclosures, each representing a different feeding treatment. Bees were fed 5 ml of 50% sucrose solution in troughs placed in each mini-hive. The four treatments were: 1) sucrose solution only (untreated control), 2) Mixture I (200 µg each of five dsRNAs added to the sugar solution), 3) Mixture II (200 µg each of 14 dsRNAs added to the sugar solution), and 4) dsRNA-GFP (200 µg dsRNA) serving as an inert dsRNA control. Mini-hives that fully consumed the treatment solutions were supplemented with candy (67% sugar powder and 33% honey). In addition, the bees were routinely fed pollen patties (70% pollen and 30% sugar powder). Each replicate of the experiment lasted for 60 days (Figure 4). Bees in each treatment were fed the respective solution daily for the first 10 days and for the last 14 days, and twice a week in the interim. *Varroa* mites were introduced into each mini-hive from day 7 till day 14. In the first replicate, 30 mites were introduced into each mini-hive; in the latter two replicates, 100 mites were introduced into each mini-hive. On day 60, all mature bees were collected, counted and shaken with 70% ethanol overnight in order to collect and count *Varroa* mites that fell off the bees. All capped brood cells were opened to collect and count *Varroa* mites. We calculated mites per bee (mature and developing). *Varroa* mites, adult bees, emerging bees and pupae were stored for molecular analyses.

### Statistical analysis

Statistical analyses were conducted with JMP statistical software version 9 (SAS Institute, Cary, NC, USA). Statistical significance was set at *P*<0.05. To test for significant differences in relative expression, one-way ANOVA was conducted on ddCt values [39]. Treatment was the main factor. To test for differences in *Varroa* mite population, two-way ANOVA was conducted on numbers of *Varroa* per bee in a block design with treatment as main effect and experimental replicate as block. To test for differences in total bee population, a similar two-way ANOVA was conducted on the total number of bees (capped brood and adults). Significant differences between treatments were tested by the Tukey-Kramer (HSD) test.

## Supporting Information

Figure S1 **DsRNA stability in the hive.** RT-PCR analysis of dsRNA-GFP stability in 50% sucrose solution under hive conditions. The solution was introduced to bees. Numbers represent days from the time of dsRNA-GFP introduction. SM = size markers.
(TIF)

Figure S2 **An example of probe determination for pre-selected genes.** **A**. Example sequences of mRNA for α tubulin of mites and insects were aligned, a conservative region was selected and a probe for selecting the homologous *Varroa* gene was determined. **B**. The determined sequence of the selected α tubulin probe based on the alignment of the known insect and mite sequences (shown in A). Alternating bases (in parentheses) were inserted to accommodate all possible sequence permutations.
(TIF)

Table S1 **DsRNA-GFP sequence used as reporter sequence and Varroa dsRNA sequences for Varroa gene silencing.**
(DOC)

Table S2 **An example of determining whether a selected** ***Varroa*** **sequence may potentially off-target bee or human gene.** Blast analysis indicated that no homology longer than 19 bases was found between this *Varroa* sequence and any bee or human sequence. An example of the longest found homology (sequence 12 of Table S1) is presented.
(DOC)

Table S3 **List of primers for dsRNA preparation.** Bold letters indicate sequences of T7 promoters. Amplicon sizes excluded T7 promoter sequences. * Designation of *Varroa* sequences as per Table S1.
(DOC)

Table S4 **List of primers and probes used for real-time, semi-quantitative RT-PCR assays and Northern blot assay.** * Designation of *Varroa* sequences as per Table S1.
(DOC)
