# Supplementary figures and images for: Bidirectional Transfer of RNAi between Honey Bee and Varroa destructor: Varroa Gene Silencing Reduces Varroa Population
Source: PLoS Pathog. 2012 Dec 20;8(12):e1003035. doi: 10.1371/journal.ppat.1003035 (PMC3534371; doi:10.1371/journal.ppat.1003035)

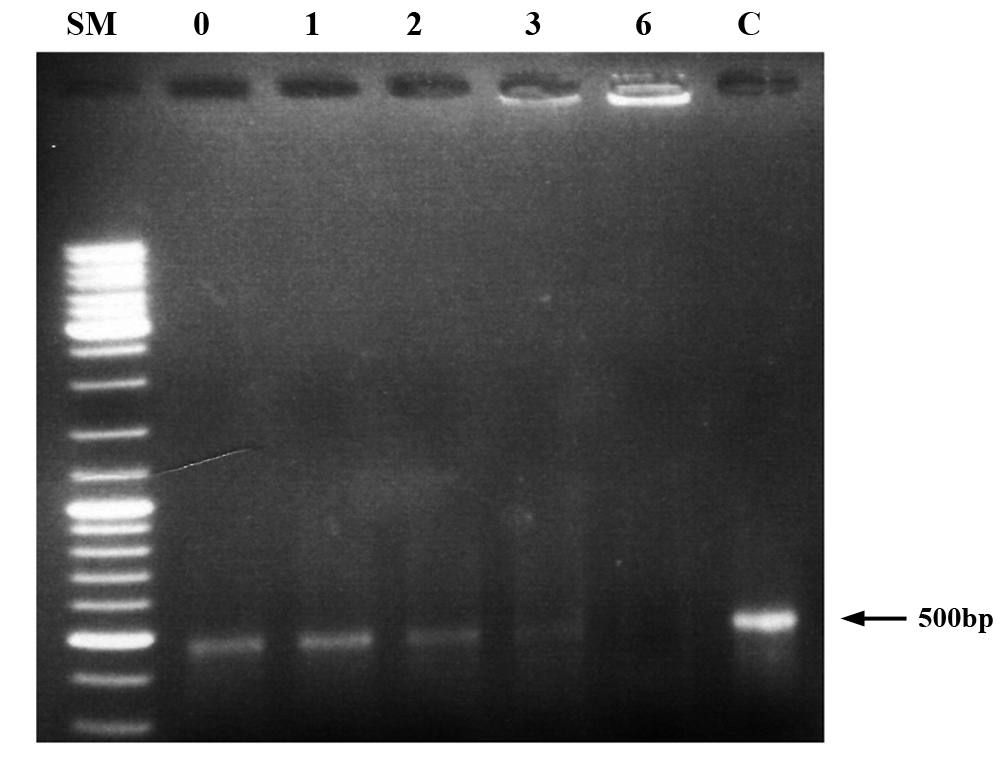

Supplement: Figure S1 — DsRNA stability in the hive. RT-PCR analysis of dsRNA-GFP stability in 50% sucrose solution under hive conditions. The solution was introduced to bees. Numbers represent days from the time of dsRNA-GFP introduction. SM = size markers. (TIF) [file ppat.1003035.s001.tif]

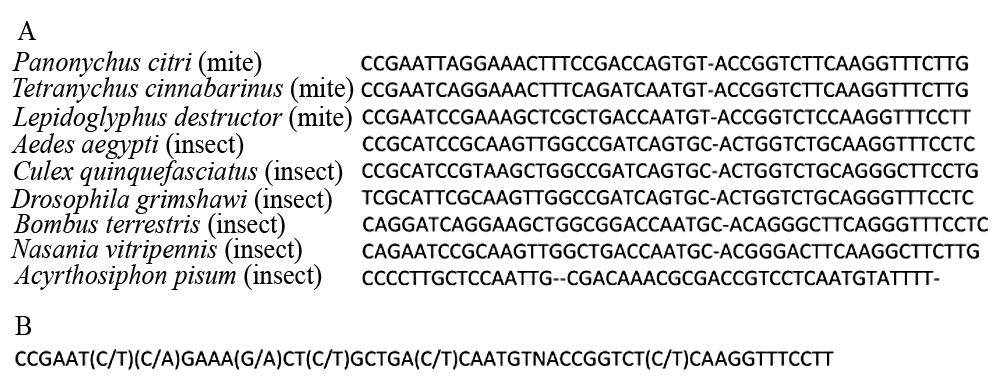

Supplement: Figure S2 — An example of probe determination for pre-selected genes. A. Example sequences of mRNA for α tubulin of mites and insects were aligned, a conservative region was selected and a probe for selecting the homologous Varroa gene was determined. B. The determined sequence of the selected α tubulin probe based on the alignment of the known insect and mite sequences (shown in A). Alternating bases (in parentheses) were inserted to accommodate all possible sequence permutations. (TIF) [file ppat.1003035.s002.tif]
