# Supplementary material for: Bidirectional Transfer of RNAi between Honey Bee and Varroa destructor: Varroa Gene Silencing Reduces Varroa Population
Source: PLoS Pathog. 2012 Dec 20;8(12):e1003035. doi: 10.1371/journal.ppat.1003035 (PMC3534371; doi:10.1371/journal.ppat.1003035)
Supplement: Table S1 — DsRNA-GFP sequence used as reporter sequence and Varroa dsRNA sequences for Varroa gene silencing. (DOC) [file ppat.1003035.s003.doc]

## Table S1. DsRNA-GFP sequence used as reporter sequence and Varroa dsRNA sequences for Varroa gene silencing

## Bold letters indicate sequences of the opposing T7 promoters.

A segment of GFP sequence, 432 -b

**TAATACGACTCACTATAGGGCGA**GCCAACACTTGTCACTACTTTCGGTTATGGTGTTCAATGCTTTGCGAGATACCCAGATCATATGAAACAGCATGACTTTTTCAAGAGTGCCATGCCTGAAGGTTATGTACAGGAAAGAACTATATTTTTCAAAGATGACGGGAACTACAAGACACGTGCTGAAGTCAAGTTTGAAGGTGATACCCTTGTTAATAGAATCGAGTTAAAAGGTATTGATTTTAAAGAAGATGGAAACATTCTTGGACACAAATTGGAATACAACTATAACTCACACAATGTATACATCATGGCAGACAAACAAAAGAATGGAATCAAAGTTAACTTCAAAATTAGACACAACATTGAAGATGGAAGCGTTCAACTAGCAGACCATTATCAACAAAATACTCCAATTGGCGATGGCCCTGTCCTTTTACCAGACAACCATTACCT**TCGCCCTATAGTGAGTCGTATTA**

**Cytoskeleton genes.**

*Varroa* sequence # 1: Similar to α-tubulin, 411b C**TAATACGACTCACTATAGGGCGA**ATGGAGAACATCGCACAGGACTTCGGTAAAAAGTGCCGATTGGGCTTCGCCATCTACCCGGCTCCGCAGGTTTCCACTGCCGTTGTCGAACCATACAACTCGGTTTTGACGACACATGCCACCCTCGAACACGCTGACTGCGTATTCATGATGGATAATGAGGCGATCTATCAGATCTGTCGTCGGAATCTTGGAGTTGAACGACCGGCGTATCAAAATCTCAATCGACTGATTAGCCAGGCCGTTTCGGCGATAACCGCTTCTCTACGTTTTTCCGGAGCGTTGAATGTTGACCTCAACGAATTTCAGACGAATCTCGTCCCCTACCCGCGAATCCATTTCCCGCTCGTCACTTATGCTCCGATTATTTCGGCTGAGAAGGCTCATCACGAGCAACATAACGTACTGGAA**TCGCCCTATAGTGAGTCGTATTA**G

*Varroa* sequence # 2: Similar to α-tubulin, 277b C**TAATACGACTCACTATAGGGCGA**GGTCTTGACAACACATGCTACCCTCGAACACGCCGACTGCGTCTTCATGATGGACAATGAGGCCATCTATCAGATCTGCCGTCGGAACCTTGGAGTCGAGCGACCGGCGTACCAGAATCTCAACCGTCTGATCAGTCAGGCCGTTTCGGCGATTACCGCTTCTCTACGTTTCTCCGGAGCGCTGAATGTTGATCTTAACGAGTTCCAAACTAATTTAGTTCCATACCCGCGAATCCATTTTCCCCTCGTCACTTACGCTCCGATCATTTCTGCTGAG**TCGCCCTATAGTGAGTCGTATTA**G

*Varroa* sequence # 3: Similar to α-tubulin, 329b C**TAATACGACTCACTATAGGGCGA**AACGCTGTGCTTCACGTAGACTCCACGTTCGAAAATGTCGACTGCACGTTTATGGTTGATAATCAAACACTCTTCAAGCTTTGTCGAGACCGGCTAAAGATTAGGAGTCCATCTTATGACAACGCAAATGCTGTCATTTCCCAGGGTTTTTCGTCAATCATGAATTCGGTGGGGCTGGATGGATCCTTGAATGTGGACCTCAGCGAGTTCCAAACAAATCTCGTCCCTTTTGGAAGATTACATTTTACGATGATGAGCTACAGTCCATTCGTTACATCCGGACACCGCGATCTAAGCCGTGAGACGTCCGTCGTGGAGATTACTCGTGA**TCGCCCTATAGTGAGTCGTATTA**G

**RNA polymerase genes**

*Varroa* sequence # 4: Similar to RNA polymerase III, 380b C**TAATACGACTCACTATAGGGCGA**TCAGATGATTGGAACGGAGGAAAATGTCCAAGTAGCATTCGTGGGCTCGATTGTCGAGTGTCACAAGCTCAAGGTGTTTACTCAGGAAGAAGCACTGAGATTCCTTGCGGCAAAGATGAAGCAGCGGATGTTTGGACCACAGAAAGCGGAAGACCCCTTGACAAGGCATGGGAAGCCGTACTTTCATCCGTAGTCAACCATATTCCCGTTCAATCGCCTGACTACAATATGACTGTCCGGGCACACTATCTTGCACTAATGGTGCGTCGCATCATTCAGGCGCGTTATGATCGCCGCTTCATTGACGATCGCGACTATTACGGCAACAAACGAATTGAGCTTCCGGGTTCGATGATATCGCTGCTGTTTGAAGACCTGTT**TCGCCCTATAGTGAGTCGTATTA**G

*Varroa* sequence # 5: Similar to RNA polymerase III, 426b C**TAATACGACTCACTATAGGGCGA**TCAATTCGTCTGCAGATCTCACCGATTTTCTGATATCGCTGGGAGTCCAGGATATTCGACTACTATGCGGAGCTGAATTCAGCAAAACACACGTCTACTATGTATTCCACAACGGTGTTATTAAAGGCGTCGTTGAGGATCATCGCAGGCTTATCAACGAGATTCGGCAATTTCGTCGGAAGGGATACTTGTCGCCTTACTTATCAGTTTATCCAAATCATCTACATCGCTGTGTGTATATTGTAACTGACGGTGGTCGTTTCTGCAGGCCGTTTATCATTGTTGAGGATGGTCAGCCAAAAGTTACGCAGAAACATTTGGACGACCTCAAAGCCAATATATATAACTTCCAAGACTTCCTGGACATGGGCTTTGTAGAGTTTCTCGATGTAAATGAGGAAAACGACGCGCTTATCGCCATTTATG**TCGCCCTATAGTGAGTCGTATTA**G

*Varroa* sequence # 6: Similar to RNA polymerase II, 366b

C**TAATACGACTCACTATAGGGCGA**AATGAGTGTTGAGCGCGGATTTAAGGCCGGTGTAGTATATAAAACAGAAACGATCAATTTGCGTAAGTTATCTGGGGATGTGGGAGTCCAGACATCGTGCGTTTTTGGTCGAAAGGCAGGAGATTCTGAGTTACAGAAATTTGTAGATGTTGATGGCCTGCCATACATCGGCAGCAGGGTAGTACAGGGAGATCCGGTATGTGCATATATAAATTTGACCACGGGACAACTGAAGACTGTAAGGTATTACTCGACCGAGCCAGCAATCGTGCATGAAGTGAAAATTCTTGGTAATGATTCCGGTACAGACACCCTCCAACAAATCCAGTTGACGTATCTTATTGATCGAACGCCAAATGATCGGAG**TCGCCCTATAGTGAGTCGTATTA**G

*Varroa* sequence # 7: Similar to RNA polymerase I, 324b

C**TAATACGACTCACTATAGGGCGA**AGGTGACATCCGTGTTCGCCGTGTACGGCATCAAAGTGGATCCAAGACATCTAAGTCTGGTAGGGGACTACATGACTTTCGACGGAGCTTACCGCGCCTTCAACAGAATCCACATGGCAAACAATGCATCGCCACTCCAGCAGATGAGCTTTGAAACGACGTGCACATTTATGAAAAACGCTGCTTTATTTGGTACGAAATCCCCTAAGACAGATACGAAGACAATCTTTGCCATGCTAATAGTGTTTCTGTTTTTAGTGCCTGGTACGATCATTAATTACGGCGTTGAAAGTAACTCCAAACAGCGACCCTATATGTCTTCAT**TCGCCCTATAGTGAGTCGTATTA**G

**Energy-related genes**

*Varroa* sequence # 8: Similar to vacuolar translocating ATPase, 311b

C**TAATACGACTCACTATAGGGCGA**CTGTACAGGGTCCGAATATAAAACTTCATACATTCAAAATCACGTATCAGGATTATGCTAAACATCGCACCATAAAAATCTTCACTAAAGTTATTTTACGCTTCAGGATAGTGGTCCGTTATGAGTGTTGCGGTATTAGTGCGTTTACAAATTTGCTAACGATATTAACAAGCTTATTTCACTCGTTGGCAGGTTTTCTAGAACGCGAGGTGAGGAAGGATAACCTTCCGATGATGTCATTCGGCGACAATCCTGAGGCGCCTCAGCCTCGGGAGATGATTGATCTAGAAGCAACCTTTGAGAAACTCGAA**TCGCCCTATAGTGAGTCGTATTA**G

*Varroa* sequence # 9: Similar to vacuolar proton ATPase, 201b

C**TAATACGACTCACTATAGGGCGA**CAATTGAATATGGACGTCACTCAGAAGTGTCTTATTGCCGAATGCTGGATTCCTGATCGCGATGTAGCAAAGGTACAAGCTGCCCTGCGACGTGGAACGGAAGCGGCTGGAAGCAGCTTCCCGTGTATCATTAACCGGTTGGAAACGGACCAAGCTCCACCGACGTTCTACAGAACGAACTCGTTTACTGCTGGCTTTCAA**TCGCCCTATAGTGAGTCGTATTA**G

*Varroa* sequence # 10: Similar to Na+/K+ ATPase, 307b

C**TAATACGACTCACTATAGGGCGA**CATCATCTTCTTCATCTGCTTGGCGGCATTCTGGACGGTTATGCTGGTCATCTTCTATCAGACACTCGATGCCTTCCAGCCAAAGTGGACCCTGGACGCTAGTCTCATTGGCACTGTACCGGGATTAGGCTTCAGGCCACGCCCACCGCTGTCTAACATCGACTCAACACTCATCTATTTCAAGGTATCTAAGCCGTTAGTGTATATGTTATATTATAGCGCTCTTTGTTATGTGGAAAGACGCCAGGGCGCGTATCTATATGGTGGTTTTCATACCAACCGTGGGAACC**TCGCCCTATAGTGAGTCGTATTA**G

**Apoptosis inhibitors**

*Varroa* sequence # 11: Similar to apoptosis inhibitor IAP, 263b

C**TAATACGACTCACTATAGGGCGA**AATGGTTTCTGCTACCTGTGAGGATAGTATGCGGGATGCTTGTATTCGTTTTCTTGCCTCGAAAGTCAATCTCAAAGCGCTTGACAGTGAGACAGAGCTTATGCTCATTGAAGAGGCCGGCAAAGTGGCAGCCCTCGTCGGTGGAGAGGAGTTTGTGCTGCTGGTTAAGCTCCTCAATTCATTAAAGGTAGATTGTACATTTTGGCGTCTTCTCGAACAAGTTAGAATCTATTTAGCAAAGTGCCAATGTATCAGCTTCCAAT**TCGCCCTATAGTGAGTCGTATTA**G

*Varroa* sequence # 12: Similar to apoptosis inhibitor FAS, 277b

C**TAATACGACTCACTATAGGGCGA**TGGCTAATTAATAGTAGGCCGAAGAACTTTTTGAGTGGCCTCGATATGTCCGACGTTGTGGCTTCGTGGGAGGTTCCTTTGGTTGGCCAAGCTTACCGAGTCGAATTCGAACACGGAAGTGCAACGGGTAAACGTGTTGTGTACGTTAATGGACTCGAGGTGTTACGAAAACACTGGCTTTTTAAGCTTGTTGGCGAGGAAAGCTTTGACATATTGGGACATAAGTGCATCATTTCTATCAAAGCCGTAGGAGGCTTCAGGTTGGTAGCAAACTCCA**TCGCCCTATAGTGAGTCGTATTA**G

*Varroa* sequence # 13: Similar to apoptosis inhibitor iap1 and iap2, 263b

C**TAATACGACTCACTATAGGGCGA**AGCCGGCTTCTTCTTCCTTGGCATGCACGATTACACGAAATGCTTCCATTGCGACGGCGGTCTGTGTAATTGGGAGACAGGTGACGACCCCTGGGTAGAGCATGCCCGCTGGTTCCCTGAATGTCAATTCGTTCAGCTAAGCAAGGGCGGAGCATTCATCGCTGAGTGCCAACAACGTCACGAAAAACTAGTTAATGGCGCGGTAGCCCAGGCAGAACTTCAGGCTTTTAGTGAAGTAGAACCGGGAGGAACAGGCAGTGACT**TCGCCCTATAGTGAGTCGTATTA**G

*Varroa* sequence # 14: Similar to apoptosis inhibitor iap1 and iap2, Reverse orientation, 282b C**TAATACGACTCACTATAGGGCGA**TTCCGCTTCATTTGAGAACTGAGCTTGAAGAAATAATGCAGTCGCCCGTCGTCAAGTTCTACCTCGAGAAAGGTGTACCGAAACAAGTGATTCGAATGACCGTAAAAAATATATGCTTGACAACGAGCGCGGTTTCCGTGATCTTGACGAAATTACACACGTACTCGGACAGGTGCTCAGCTTCGGCAACAAGAAGACTGCGCCTGCCAATGAAAAAGGTAGGTGGATACCGGATATTTGTCGGGAATTCAATGCAGCTGAACCCGATGAGGTTGATTCAGA**TCGCCCTATAGTGAGTCGTATTA**G
