# Supplementary material for: Bidirectional Transfer of RNAi between Honey Bee and Varroa destructor: Varroa Gene Silencing Reduces Varroa Population
Source: PLoS Pathog. 2012 Dec 20;8(12):e1003035. doi: 10.1371/journal.ppat.1003035 (PMC3534371; doi:10.1371/journal.ppat.1003035)
Supplement: Table S2 — An example of determining whether a selected Varroa sequence may potentially off-target bee or human gene. Blast analysis indicated that no homology longer than 19 bases was found between this Varroa sequence and any bee or human sequence. An example of the longest found homology (sequence 12 of Table S1) is presented. (DOC) [file ppat.1003035.s004.doc]

**Table S2: An example of determining whether a selected *Varroa* sequence may potentially off-target bee or human genes.**

| Organism database | Accession number | Identity | alignment |
| --- | --- | --- | --- |
| *A. mellifera* | XM_395449.3 | 15/15 | 20 CGAAGAACTTTTTGA 34  |||||||||||||||  428 CGAAGAACTTTTTGA 442 |
| *A. mellifera* | XM_393956.3 | 15/15 | 20 CGAAGAACTTTTTGA 34  |||||||||||||||  1139 CGAAGAACTTTTTGA 1153 |
| *A. mellifera* | XM_625209.2 | 15/15 | 115 GGAAGTGCAACGGGT 129  |||||||||||||||  2727 GGAAGTGCAACGGGT 2741 |
| *A. mellifera* | XM_001120588.1 | 15/15 | 190 GTTGGCGAGGAAAGC 204  |||||||||||||||  9171 GTTGGCGAGGAAAGC 9185 |
| *A. mellifera* | XM_623599.2 | 15/15 | 221 ATAAGTGCATCATTT 235  |||||||||||||||  171 ATAAGTGCATCATTT 157 |
| *H. sapiens* | NT_007933.15 | 19/19 | 261 GTTGGTAGCAAACTCCAGT 279  |||||||||||||||||||  28654284 GTTGGTAGCAAACTCCAGT 28654302 |
| *H. sapiens* | NW_001839064.2 | 19/19 | 261 GTTGGTAGCAAACTCCAGT 279  |||||||||||||||||||  8223222 GTTGGTAGCAAACTCCAGT 8223204 |

Blast analysis indicated that no homology longer than 19 bases was found between this *Varroa* sequence and any bee or human sequence. An example of the longest found homology (sequence 12 of Table S1) is presented.
