# Supplementary material for: Bidirectional Transfer of RNAi between Honey Bee and Varroa destructor: Varroa Gene Silencing Reduces Varroa Population
Source: PLoS Pathog. 2012 Dec 20;8(12):e1003035. doi: 10.1371/journal.ppat.1003035 (PMC3534371; doi:10.1371/journal.ppat.1003035)
Supplement: Table S3 — List of primers for dsRNA preparation. Bold letters indicate sequences of T7 promoters. Amplicon sizes excluded T7 promoter sequences. * Designation of Varroa sequences as per Table S1. (DOC) [file ppat.1003035.s005.doc]

**Table S3: List of primers for dsRNA preparation**

Bold letters indicate sequences of T7 promoters. Amplicon sizes excluded T7 promoter sequences

| **Sequence*** | **Primers** | **Amplicon (bp)** |
| --- | --- | --- |
| *Varroa* sequence # 1 | F: 5' C**TAATACGACTCACTATAGGGCGA**ATGGAGAACATCGCACAG 3'  R: 5' C**TAATACGACTCACTATAGGGCGA**TTCCAGTACGTTATGTTGCTC 3' | 411bp |
| *Varroa* sequence # 2 | F :5' C**TAATACGACTCACTATAGGGCGA**GGTCTTGACAACACATGCTAC 3'  R :5' C**TAATACGACTCACTATAGGGCGA**CTCAGCAGAAATGATCGG 3' | 277bp |
| *Varroa* sequence # 3 | F: 5' C**TAATACGACTCACTATAGGGCGA**AACGCTGTGCTTCACGTA 3'  R: 5' C**TAATACGACTCACTATAGGGCGA**TCACGAGTAATCTCCACGA 3' | 329bp |
| *Varroa* sequence # 4 | F: 5' C**TAATACGACTCACTATAGGGCGA**TCAGATGATTGGAACGGA 3'  R: 5' C**TAATACGACTCACTATAGGGCGA**AACAGGTCTTCAAACAGCAG 3' | 380bp |
| *Varroa* sequence # 5 | F: 5' C**TAATACGACTCACTATAGGGCGA**TCAATTCGTCTGCAGATCTC 3'  R: 5' C**TAATACGACTCACTATAGGGCGA**CATAAATGGCGATAAGCG 3' | 426bp |
| *Varroa* sequence # 6 | F: 5' C**TAATACGACTCACTATAGGGCGA**AATGAGTGTTGAGCGCGG 3'  R: 5' C**TAATACGACTCACTATAGGGCGA**CTCCGATCATTTGGCGTT 3' | 366bp |
| *Varroa* sequence # 7 | F: 5' C**TAATACGACTCACTATAGGGCGA**AGGTGACATCCGTGTTCG 3'  R: 5' C**TAATACGACTCACTATAGGGCGA**ATGAAGACATATAGGGTCGCT 3' | 324bp |
| *Varroa* sequence # 8 | F: 5' C**TAATACGACTCACTATAGGGCGA**CTGTACAGGGTCCGAATATAAA 3'  R: 5' C**TAATACGACTCACTATAGGGCGA**TTCGAGTTTCTCAAAGGTTG 3' | 311bp |
| *Varroa* sequence # 9 | F:5' C**TAATACGACTCACTATAGGGCGA**CAATTGAATATGGACGTCACTC 3'  R: 5' C**TAATACGACTCACTATAGGGCGA**TTGAAAGCCAGCAGTAAACG 3' | 201bp |
| *Varroa* sequence # 10 | F: 5' C**TAATACGACTCACTATAGGGCGA**CATCATCTTCTTCATCTGCTTG 3'  R: 5' C**TAATACGACTCACTATAGGGCGA**GGTTCCCACGGTTGGTAT 3' | 290bp |
| *Varroa* sequence # 11 | F: 5' C**TAATACGACTCACTATAGGGCGA**AATGGTTTCTGCTACCTGTG 3'  R: 5' C**TAATACGACTCACTATAGGGCGA**ATTGGAAGCTGATACATTGG 3' | 263bp |
| *Varroa* sequence # 12 | F: 5' C**TAATACGACTCACTATAGGGCGA**TGGCTAATTAATAGTAGGCCG 3'  R: 5' C**TAATACGACTCACTATAGGGCGA**TGGAGTTTGCTACCAACCT 3' | 277bp |
| *Varroa* sequence # 13 | F: 5' C**TAATACGACTCACTATAGGGCGA**AGCCGGCTTCTTCTTCCT 3'  R: 5' C**TAATACGACTCACTATAGGGCGA**AGTCACTGCCTGTTCCTCC 3' | 263bp |
| *Varroa* sequence # 14 | F: 5' C**TAATACGACTCACTATAGGGCGA**TTCCGCTTCATTTGAGAAC 3'  R: 5' C**TAATACGACTCACTATAGGGCGA**TCTGAATCAACCTCATCGG 3' | 282bp |
| Segment of GFP | F: 5' **TAATACGACTCACTATAGGGCGA**GCCAACACTTGTCACTACTAGAAAGAGAA 3'  R: 5' **TAATACGACTCACTATAGGGCGA**AGGTAATGGTTGTCTGGTAAAGGAC 3' | 432 bp |

*Designation of *Varroa* sequences as per Table S1.
