# Supplementary material for: Bidirectional Transfer of RNAi between Honey Bee and Varroa destructor: Varroa Gene Silencing Reduces Varroa Population
Source: PLoS Pathog. 2012 Dec 20;8(12):e1003035. doi: 10.1371/journal.ppat.1003035 (PMC3534371; doi:10.1371/journal.ppat.1003035)
Supplement: Table S4 — List of primers and probes used for real-time, semi-quantitative RT-PCR assays and Northern blot assay. * Designation of Varroa sequences as per Table S1. (DOC) [file ppat.1003035.s006.doc]

**Table S4: List of primers and probes used for real-time, semi-quantitative RT-PCR assays and Northern blot assay.**

| **Sequence*** | **Primers** | **Probe** | **Used for** | **Amplicon (bp)** |
| --- | --- | --- | --- | --- |
| *Varroa* sequence # 4. | F: 5' AAAGGGCAGGTGCTTATCAA 3'  R: 5' TGTCCAGGGTCGAGAGTAGC 3' | Roche # 25 | Real-time PCR | 65 |
| *Varroa* sequence # 9. | F: 5' ACCTTTTTCAAAGACCGAACC 3'  R: 5' CGAAGACTCCGTTCGAAAAC 3' | Roche # 101 | Real-time PCR | 62 |
| *Varroa* sequence # 14. | F: 5' CTAGTTAATGGCGCGGTAGC 3'  R: 5' TCCTCCCGGTTCTACTTCAC 3' | Roche # 124 | Real-time PCR | 63 |
| *Varro*a 18S RNA | F: 5' AATGCCATCATTACCATCCTG 3'  R: 5' CAAAAACCAATCGGCAATCT 3' | Roche # 153 | Real-time PCR | 60 |
| *Varroa* sequence # 12 | F: 5' ATCTGCCCACGTCAGCGTTT 3'  R: 5' GTCCGTCATTTCGGCTTTGG 3' |  | Semi-quantitive PCR | 317 |
| *Varroa* Actin | F:5' AAGTCGTACGAGCTTCCCGAC 3'  R:5' ACAGGGAGGCAAGGATGGAAC 3' |  | Semi-quantitive PCR | 336 |
| Segment of GFP | F: 5' gccaacacttgtcactactttctctt 3'  R: 5' aggtaatggttgtctggtaaaaggac 3' |  | Northern blot | 432 |

* Designation of *Varroa* sequences as per Table S1.
